# Existence of gender bias in clinical dentistry: A cross-sectional study on Perception and experience of dental clinicians at a private dental institute in Lahore

**DOI:** 10.12669/pjms.42.5.13623

**Published:** 2026-05

**Authors:** Muhammad Abdullah, Mahnoor Sikandar, Kanza Iqbal, Gul E Zahra

**Affiliations:** 1 Muhammad Abdullah House Officer, CMH Lahore Medical College and Institute of Dentistry, Lahore Pakistan; 2 Mahnoor Sikander Senior Demonstrator,Department of Operative Dentistry & Endodontics, CMH Lahore Medical College and Institute of Dentistry, Lahore Pakistan; 3 Kanza Iqbal Senior Registrar,Department of Operative Dentistry & Endodontics, CMH Lahore Medical College and Institute of Dentistry, Lahore Pakistan; 4 Gul E Zahra 4^th^ Year BDS, CMH Lahore Medical College and Institute of Dentistry, Lahore Pakistan

**Keywords:** Dentistry, Dentists, Patients, Professional Practice, Prejudice, Sexism

## Abstract

**Background & Objective:**

Gender bias continues to shape the experiences of healthcare professionals, including those in dentistry. Although more women are joining the dental workforce, unequal opportunities, differences in patient perceptions, and subtle workplace discrimination still exist. This study explored dental clinicians’ perceptions and experiences concerning gender-bias in clinical opportunities and patient preferences.

**Methodology:**

A cross-sectional survey was conducted among 110 dental clinicians at CMH Lahore Medical College & Institute of Dentistry from April-August 2025. Data was collected using a validated questionnaire (Cronbach’s α = 0.739) and analyzed through descriptive statistics and chi-square tests using SPSS version 26.

**Results:**

Out of 110 respondents, 70% were female and 30% male. Significantly more females than males (57.1% vs. 3%, p<0.001) felt that the opposite gender was more confident clinically, felt males were generally more favored in dentistry (75.3% vs. 24.2%, p<0.001), reported facing some form of gender-based discrimination in terms of clinical opportunities or recognition of their skills(61.0% vs. 12.1%, p<0.001), and experienced patients requesting to be treated by the opposite gender (68.8% vs. 42.4%, p=0.009).

**Conclusion:**

The study highlights that gender bias in dentistry remains a subtle but influential factor in shaping clinical experiences and confidence levels. Although many patients say that they have no gender preferences upfront but gender biases are still evident. Although patient education can help but cultural mindsets are not easy to change. However, encouraging mentorship, promoting gender-sensitive training, and ensuring fair institutional policies can help create a more balanced and supportive professional environment for all dental practitioners and reduce interpersonal bias.

## INTRODUCTION

At birth a person is either a Male or a Female, which is assigned as their gender. In better understanding, gender is a social construct in which roles and behaviours are segregated for men and women. In today’s world, gender is an important factor in all sorts of human activities including healthcare.^1^ Characteristics such as gender, ethnicity and disabilities greatly influence the recruitment of personnels in dentistry along with impacting the perceptions about suitability of the candidates, thus creating a bias.^2^ Unfortunately, prejudice based on gender is a common problem in the healthcare system and these gender biases can cause great losses for an individual in the healthcare in terms of opportunities and resources.^3^ Healthcare professionals should treat all their patients equally irrespective of their gender.^4^ Although many patients report no preference, an issue of gender-based preference of doctors does exist.^5^

When it comes to gender biases in dentistry, it seems like mostly women are the ones at a disadvantage. Especially in underdeveloped nations such as India and Pakistan, the growth of women in the dental field has been stagnant.^6^ Over the past years, Dentistry has had enrollment of more female students throughout the world. Despite this shift, gender disparities still exist in areas such as leadership opportunities, academic advancement, procedural allocation and decision-making authority. Even though women make up a large proportion of dental workforce worldwide, they still face underrepresentation at senior positions.^3^,^7^,^8^ Various studies concluded that male dentists are seen as more technically skilled and authoritative while female dentists are seen as empathetic, better at communication and more suitable for pediatric or preventive care.^9^

In Pakistan and adjacent countries, gender plays an important role in career trajectories. Studies from Pakistan have reported gender inequalities in academic leadership within medical and dental institutions with males having more senior positions despite significant female participation at junior level.^10^

Patient-driven gender bias is another aspect in clinical dentistry. Some studies from South Asia concluded that patients had preferences for healthcare providers based on gender, especially for invasive procedures and culturally sensitive ones.^11^-^13^

Although several studies have examined patient preferences in dental care or medical doctors’ perceptions of gender bias within Pakistan, limited research has focused on the perspectives and lived experiences of dental clinicians themselves. Hence, the purpose of this study was to explore dental clinicians’ perceptions and experiences concerning gender-bias in clinical opportunities and patient preferences.

Understanding clinicians’ perceptions is essential, as perceived bias may influence job satisfaction, professional identity formation, self-confidence, and career progression. Furthermore, identifying whether gender-based disparities exist in clinical opportunities, patient interactions, or recognition of skills can inform institutional policies aimed at promoting equity.

## METHODOLOGY

This was a descriptive cross-sectional study in which data was collected using convenience sampling from dental clinicians working at CMH Lahore Medical College & Institute of Dentistry. The duration of the study was from April to August 2025. The following formula was used to calculate sample size:


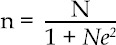


Sample size (n) of 110 dental clinicians was calculated with population size (N)=150. Marginal error (e) was kept at 0.05.^14^

### Ethical approval:

This study was approved by the ethical review committee at CML LMC & IOD (No: 113/ERC/CMH/LMC Date: February 20, 2025). Written informed consent was obtained from all participants prior to data collection.

### Inclusion criteria:

- Dental clinicians working at CMH Lahore Institute of Dentistry (including house officers).
- All those who consented to participate.
- Both genders.
- All ages.

### Exclusion criteria:

- Clinicians at any other teaching hospital or private clinics than CMH LMC.
- Any person who did not consent to take part in the research.

### Data collection methods:

The study tool used was a questionnaire designed on ‘Google forms’. Social media platforms including WhatsApp was used to contact the clinicians to collect their responses using the online link for the questionnaires. Those who did not respond via the links were approached with hard copies of this questionnaire to collect their responses.

The first part of the form briefly mentioned the purpose of the study and asked for the consent of the participants. It was mentioned that the participation was voluntary, no identification information was required and the responses will be kept confidential and only be used for research purposes. Section two asked for demographic details such as age, gender and work experience etc. The third section contained questions for dental clinicians in order to assess the existence of gender biases in their perspectives along with any biases that were observed by the clinicians throughout their work experience. Questionnaire was screened and evaluated by five subject experts in their respective fields to check its validity. Reliability was calculated as follows:

**Table:** 

Reliability Statistics		
Cronbach’s Alpha	Cronbach’s Alpha Based on Standardized Items	N of Items
0.739	0.768	11

### Statistical Analysis:

Descriptive statistics including frequency and percentages for qualitative data whereas mean and S.D for quantitative data was calculated. For the significance, Chi-square test was applied. SPSS version 26 was used, and all results were analysed at 95% confidence level.

## RESULTS

The questionnaire was filled out by 110 dental clinicians. Demographic information is given in Tables-I and II. The participants included 70% females and 30% males. About 34.5% belonged to the age group of 21-25 years, 43.6% were between 26-30 years and 21.8% were above 30 years of age. There were 21.8% of the participants who had clinical work experience of less than a year, 31.8% had experience of 1-3 years, 20% had 3-5 years’ worth of experience and 26.4% had experience of more than five years. The participants were asked about how confident they felt in clinical settings and they scored themselves on a scale of 1-10. The mean was 8.0 (S.D 1.5, Median 8.0).

**Table-I:** Cross-tabulation of gender and age with perception and experience of gender bias. P values are from chi square and Fisher’s exact tests.

Questions about perception of gender bias		n (%)	Demographics						
			Gender			Age			
			Male	Female	p	21-25	26-30	>30	p
Distinction between clinical skills due to gender?	Yes	44 (40.0)	6 (18.2)	38 (49.4)	0.002	19 (50.0)	16(33.3)	9 (37.5)	0.282
	No	66 (60.0)	27 (81.8)	39 (50.6)		19 (50.0)	32(66.7)	15 (62.5)	
Males or Females have better clinical skills?	Males	26 (23.6)	6 (18.2)	20 (26.0)	0.109	11 (28.9)	10(20.8)	5 (20.8)	0.864
	Females	7 (6.4)	0 (0.0)	7 (9.1)		3(7.9)	3(6.3)	1(4.2)	
	Does not depend on gender	77 (70.0)	27 (81.8)	50 (64.9)		24 (63.2)	35(72.9)	18 (75.0)	
Clinical opportunities are more inclined towards one gender?	Yes	63 (57.3)	9 (27.3)	54 (70.1)	<0.001	27 (71.1)	27(56.3)	9 (37.5)	0.033
	No	47 (42.7)	24 (72.7)	23 (29.9)		11 (28.9)	21(43.8)	15 (62.5)	
Males or Females are better at communication?	Males	17 (15.5)	4 (12.1)	13 (16.9)	0.076	6 (15.8)	9(18.8)	2(8.3)	0.827
	Females	19 (17.3)	2 (6.1)	17 (22.1)		7 (18.4)	7(14.6)	5 (20.8)	
	Does not depend on gender	74 (67.3)	27 (81.8)	47 (61.0)		25 (65.8)	32(66.7)	17 (70.8)	
Male or Female dentists are favored?	Males	66 (60.0)	8 (24.2)	58 (75.3)	<0.001	27 (71.1)	26(54.2)	13 (54.2)	0.280
	Females	4 (3.6)	1 (3.0)	3 (3.9)		1(2.6)	1(2.1)	2(8.3)	
	No Favoritism	40 (36.4)	24 (72.7)	16 (20.8)		10 (26.3)	21(43.8)	9 (37.5)	
Is opposite gender more confident?	Yes	45 (40.9)	1(3.0)	44 (57.1)	<0.001	14 (36.8)	20(41.7)	11 (45.8)	0.774
	No	65 (59.1)	32 (97.0)	33 (42.9)		24 (63.2)	28(58.3)	13 (54.2)	
Patients prefer Male or Female dentists?	Males	22 (20.0)	6 (18.2)	16 (20.8)	0.217	5 (13.2)	11(22.9)	6 (25.0)	0.013
	Females	51 (46.4)	12 (36.4)	39 (50.6)		26 (68.4)	15(31.3)	10 (41.7)	
	Equal preferences	37 (33.6)	15 (45.5)	22 (28.6)		7 (18.4)	22(45.8)	8 (33.3)	
You would prefer a male of female dentist for your treatment?	Male	18 (16.4)	13 (39.4)	5 (6.5)	<0.001	5(13.2)	8(16.7)	5 (20.8)	0.835
	Female	14 (12.7)	0 (0.0)	14 (18.2)		5(13.2)	5(10.4)	4 (16.7)	
	No preference	78 (70.9)	20 (60.6)	58 (75.3)		28 (73.7)	35 (72.9)	15 (62.5)	
***Questions about experience of gender bias***									
Discriminated with regards to clinical skills and opportunities?	Yes	51 (46.4)	4 (12.1)	47 (61.0)	<0.001	21 (55.3)	20 (41.7)	10 (41.7)	0.397
	No	59 (53.6)	29 (87.9)	30 (39.0)		17 (44.7)	28 (58.3)	14 (58.3)	
Patient refused treatment and asked for a senior doctor?	Yes	53 (48.2)	7 (21.2)	46 (59.7)	<0.001	18 (47.4)	24 (50.0)	11 (45.8)	0.939
	No	57 (51.8)	26 (78.8)	31 (40.3)		20 (52.6)	24 (50.0)	13 (54.2)	
Patient requested treatment be done by opposite gender?	Yes	67 (60.9)	14 (42.4)	53 (68.8)	0.009	24 (63.2)	29 (60.4)	14 (58.3)	0.927
	No	43 (39.1)	19 (57.6)	24 (31.2)		14 (36.8)	19 (39.6)	10 (41.7)	
Total		110 (100)	33 (30.0)	77 (70.0)		38 (34.6)	48 (43.6)	24 (21.8)	

**Table-II:** Cross-tabulation of clinical experience with perceptions and experiences of gender bias. P values are from chi square and Fisher’s exact tests.

Questions about perception of gender bias		n (%)	Clinical Work Experience (years)				
			<1	1-3	3-5	>5	p
Distinction between clinical skills due to gender?	Yes	44 (40.0)	12(50.0)	13(37.1)	9(40.9)	10(34.5)	0.684
	No	66 (60.0)	12(50.0)	22(62.9)	13(59.1)	19(65.5)	
Males or Females have better clinical skills?	Males	26(23.6)	5(20.8)	10(28.6)	5(22.7)	6(20.7)	0.990
	Females	7(6.4)	2(8.3)	2(5.7)	1(4.5)	2(6.9)	
	Does not depend on gender	77(70.0)	17(70.8)	23(65.7)	16(72.7)	21(72.4)	
Clinical opportunities are more inclined towards one gender?	Yes	63(57.3)	15(62.5)	21(60.0)	13(59.1)	14(48.3)	0.714
	No	47(42.7)	9 (37.5)	14(40.0)	9 (40.9)	15(51.7)	
Males or Females are better at communication?	Males	17(15.5)	6(25.0)	4(11.4)	4(18.2)	3(10.3)	0.601
	Females	19(17.3)	3(12.5)	9(25.7)	3(13.6)	4(13.8)	
	Does not depend on gender	74(67.3)	15(62.5)	22(62.9)	15(68.2)	22(75.9)	
Male or Female dentists are favored?	Males	66(60.0)	18(75.0)	19(54.3)	13(59.1)	16(55.2)	0.643
	Females	4(3.6)	0(0.0)	1(2.9)	1(4.5)	2(6.9)	
	No Favoritism	40(36.4)	6(25.0)	15(42.9)	8(36.4)	11(37.9)	
Is opposite gender more confident?	Yes	45(40.9)	10(41.7)	13(37.1)	9(40.9)	13(44.8)	0.941
	No	65(59.1)	14(58.3)	22(62.9)	13(59.1)	16(55.2)	
Patients prefer Male or Female dentists?	Males	22 (20.0)	5(20.8)	4(11.4)	4(18.2)	9(31.0)	0.648
	Females	51 (46.4)	12(50.0)	17(48.6)	11(50.0)	11(37.9)	
	Equal preferences	37 (33.6)	7(29.2)	14(40.0)	7(31.8)	9(31.0)	
You would prefer a male of female dentist for your treatment?	Male	18 (16.4)	3(12.5)	7(20.0)	2(9.1)	6(20.7)	0.744
	Female	14 (12.7)	4(16.7)	3(8.6)	2(9.1)	5(17.2)	
	No preference	78 (70.9)	17(70.8)	25(71.4)	18(81.8)	18(62.1)
***Questions about experience of gender bias***							
Discriminated with regards to clinical skills and opportunities?	Yes	51 (46.4)	12(50.0)	16(45.7)	13(59.1)	10(34.5)	0.360
	No	59 (53.6)	12(50.0)	19(54.3)	9(40.9)	19(65.5)	
Patient refused treatment and asked for a senior doctor?	Yes	53 (48.2)	11(45.8)	15(42.9)	12(54.5)	15(51.7)	0.813
	No	57 (51.8)	13(54.2)	20(57.1)	10(45.5)	14(48.3)	
Patient requested treatment be done by opposite gender?	Yes	67 (60.9)	13(54.2)	20(57.1)	15(68.2)	19(65.5)	0.702
	No	43 (39.1)	11(45.8)	15(42.9)	7(31.8)	10(34.5)
Total		110 (100)	24(21.8)	35(31.8)	22(20.0)	29(26.4)	

Majority of the clinicians felt that there was no distinction between clinical skills based on the gender (60%), better clinical skills does not depend on the gender (70%), better communication skills does not depend on the gender (67.3%), and said that they have no gender preferences when choosing a dentist for themselves (70.9%). However, a majority felt that clinical opportunities were more inclined towards one gender (57.3%), males were more favoured in dentistry (60%) while patients preferred female dentists (46.4%). It was also reported that 40.9% felt that the opposite gender felt more confident in clinical settings while 59.1% disagreed to it.

Most clinicians did not experience any discrimination regarding clinical opportunities (53.6%) or any instance where the patient refused treatment and asked for a senior doctor (51.8%). However, about 60.9% of the clinicians experienced patients asking to be treated by the opposite gender.

**Fig.1:**
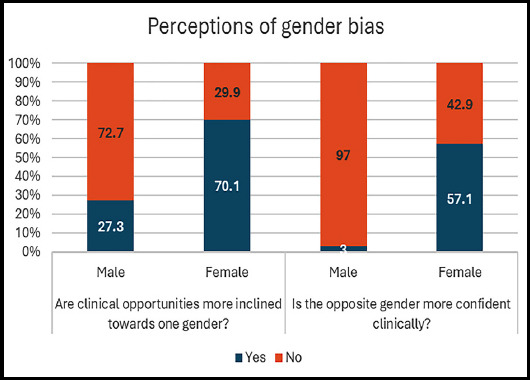
Perception of Gender Bias as felt by Males vs. Females.

**Fig.2:**
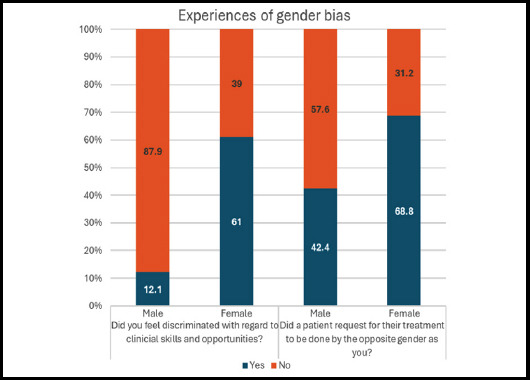
Experiences of gender bias reported by Males vs Females.

Tables-I and II display results of chi-square and Fisher’s exact tests to look for significant differences in perception and experiences between genders, age groups and years of clinical work experience. Significantly more females than males reported that they felt there was a distinction in clinical skills due to gender (49.4% vs. 18.2%, p=0.002), felt clinical opportunities were inclined towards one gender (70.1% vs. 27.3%, p<0.001, Fig 1), felt that males were favoured more in dentistry (75.3% vs. 24.2%, p<0.001), felt that the opposite gender was more confident clinically (57.1% vs. 3.0%, p<0.001, Fig 1), faced discrimination in regards to opportunities (61.0% vs. 12.1%, p<0.001, Fig 2), reported patients refusing treatment and asking for a senior doctor (59.7% vs. 21.2%, p<0.001), and experienced patients requesting for treatment to be done by the opposite gender (68.8% vs. 42.2%, p=0.009, Fig 2). However, males compared to females were significantly more likely to prefer a male dentist to get a dental treatment for themselves (39.4% vs. 6.5%, p<0.001).

The 21-25 years age group were significantly more likely to report that they felt clinical opportunities were more inclined towards one gender (71.1%, p=0.033) and that patients preferred female dentists (68.4%, p=0.013) compared to the 26-30 years and >30 years groups.

Confidence scores were not normally distributed (Kolmogorov Smirnov p<0.001) so Mann Whitney and Kruskal Wallis tests were run for comparisons between gender, age and work experience. There were no significant differences between genders and age groups in terms of confidence level of clinicians while in their practice. However, post-hoc analysis of Kruskal Wallis showed that clinicians with a work experience of <1 year had significantly lower confidence levels (Mean=7.3, SD=1.4, Median=8.0) compared to those with 3-5 years (Mean=8.6, SD=1.4, Median=8.0, p=0.003) and >5 years (Mean=8.3, SD=1.3, Median=8.0, p=0.012) of experience.

## DISCUSSION

The survey unveiled striking disparity in gender bias within clinical dentistry. While most research participants believed that female dentists are preferred more by the patients, they were also of the opinion that male dentists are more favored for skills and work opportunities. The study also revealed that in clinical settings male dentists were perceived to be more confident. A significant number of female dentists, due to their gender, reported experiencing discrimination related to their clinical skills or work opportunities. This bifocal view brings attention to the intricate nature of gender bias in dentistry, where one gender may be preferred in one area while experiencing limitations in another.

The results show both correspondence and divergence from the existing literature. A cross-sectional study from Karachi revealed that male patients preferred getting their treatment done from a male dentist while majority of female patients showed no preference.^8^ One-third of the clinicians in this study said that the patients had no preference regarding the gender of the dentist treating them while 50% said that patients preferred females dentists. However, it wasn’t found whether male or female patients had such preferences. Similarly, the patient preference for male dentists for certain procedures, and female dentists for others, aligns with a study at CMH Lahore which concluded that patients preferred female dental practitioners for pediatric and general care and male dentists for more invasive procedures like prosthetics and extractions.^15^ This notion, though not directly negative reinforces a type of gender stereotyping that suggests that patients rather than choosing their dentist on the grounds of qualification and experience depend on the idea of what each gender is best at, which limits the professional opportunities. In contrast, other studies from Pakistan, India and Saudi Arabia have demonstrated that a considerable proportion of patients do not have gender preferences at all.^15^-^17^

A higher number of participants said that males are more skilled and confident clinically while females are better at communication. These results resonate with findings of other studies in Pakistan and South Asia. For example, research found that patients often prefer male dentists for attributes like decisiveness or experience, whereas female dentists were seen as superior in traits such as sensitivity or attention to detail.^7^ In both studies, cultural and trust dimensions emerged as strong determinants of preference, highlighting that stereotype-driven judgments are embedded in broader social norms.

Another relevant study examined gender disparities in leadership in dental and medical colleges in Khyber Pakhtunkhwa, Pakistan. They found that top-tier leadership positions (e.g. deans, principals) were overwhelmingly occupied by men (84.5 %), and female leaders reported challenges in maintaining work–life balance and receiving institutional support.^18^ That pattern echoes findings of this study in which around 60% said that opportunities were more inclined towards one gender and that males were favored more in dentistry. Along with this perceived favoritism, 61% of females dentists faced discrimination with regards to opportunities in the field of dentistry.

Globally, there has been persistent underrepresentation of women in dentistry leadership, pointing to barriers such as lack of mentorship, organizational culture, and gendered expectations.^19^ Challenges also exist for women in dental research and practice, due to limited support structures, and gendered assumptions that hinder advancement.^9^ The findings of this study about discrimination and confidence align well with those challenges.

The finding that male dentists appear to be more confident and receive more opportunities corresponds to a broader trajectory. A mixed method study on academic leadership in dentistry across Pakistan emphasizes that women continue to face discrimination, limited opportunities and challenges in pertaining a balance between personal and professional life.^18^

Younger age groups felt more gender bias in terms of clinical opportunities and patient preferences. This finding is backed up by a study that claims that gender-related biases are often experienced by young clinicians and students. This in return can gravely impact their confidence and professional development.^20^ Moreover, the patient preferences for the gender of their clinicians may exaggerate the perceptions of bias among young professionals who are still trying to find their footing.^7^

Females tend to achieve higher merit, hence get admission in medical and dental colleges which accounts for more female doctors. Females make upto 70% of enrollment in dental schools of Pakistan.^18^ This study had a higher percentage of females as the sample included house officers (fresh graduates) and post graduate trainees who were mostly females, inducted because of higher merit.

### Strengths:

The strengths of this study include that it was conducted in CMH Lahore which is one of the largest private dental institutes in the city so these findings may be representative of other private dental institutes. While other studies focused on perceptions of the patients, this study directly asked the dental clinicians about their perceived gender-bias.

### Limitations:

On the other hand, the study was conducted at a single institution with a relatively modest sample size (n = 110), which may limit the applicability of the findings to broader populations. Another limitation was that convenience sampling was used, which might have introduced selection bias. The study also had an imbalance between number male and female clinicians that may have under-represented one side. This highlights the fact that future studies should be carried out using a random multi-centered sampling approach and equal male-female ratio to gain more comprehensive insights.

## CONCLUSION

The study concludes that there is a visible gender bias within dentistry which influences clinical experiences and patient preferences. While majority of participants had a point of view that skills are not gender dependent still such gender-based stereotypes prevail that continue to influence perception and practice. Dental institutions should carry out career counselling sessions, mentorship programs specifically targeting female students to assist them in managing career related challenges and to boost their confidence. Patient awareness campaigns should also be carried out to challenge gender-based stereotypes. Such policies should be implemented at professional level that ensure equal clinical training opportunities, promotions and leadership roles draws attention to urgent need for changes in mentorship and institutional policies as there is underrepresentation of women in academic leadership.

### Authors’ Contributions:

**MA:** conception and design; data acquisition, interpretation, analysis; drafted and revised the manuscript.

**MS:** Design; data acquisition, interpretation and analysis; revised the manuscript.

**KI:** Conception and design; data interpretation and analysis; Critically revised the manuscript and gave the final approval.

**GEZ:** Contributed towards data acquisition, analysis and interpretation of the results, drafting of the manuscript.
